# 

**Published:** 1983-04

**Authors:** 


Contents

51. Art and Science in Medicine
H. K. Bourns

57. Whither Midwifery?
Gordon M. Stirrat

61. Mesmerism, Galvanism and Quackery
P. S. Brown

65. Lower Limb Swelling due to Femoral Aneurysms
W. B. Campbell, N. J. McC. Mortensen, N. I. Ramus and J. H. Peacock

68. Nutritional Ricketts in Rastafarian Children
A. P. Wolinski, R. Narkielny and A. Duncan

73. Gastric Tuberculosis

M. M. Guirgis, Adly F. Ghaly and L. Abadir

76. Letter to the Editor

77. From our 'Foreign' Correspondent

80. From our Correspondents

83. West Country Physicians Club

85. South West Orthopaedic Club

90. Association of Clinical Pathologists, Wessex Branch

92. South West, Wessex and South Wales Rheumatology Club

94. Examination Results

